# COVID-19 seroprevalence in military police force, Southern Brazil

**DOI:** 10.1371/journal.pone.0249672

**Published:** 2021-04-22

**Authors:** Alessandro C. Pasqualotto, Paula de Castro Pereira, Daiane F. Dalla Lana, Alexandre V. Schwarzbold, Marco S. Ribeiro, Cezar V. W. Riche, Cristiani Pilati P. Castro, Paula L. Korsack, Paulo Emilio B. Ferreira, Guilherme de C. Domingues, Giorgia T. Ribeiro, Marcelo Carneiro, Cassia Ferreira B. Caurio, Izadora Clezar da S. Vasconcellos, Lidiana M. Knebel, Lucas Zamberlan, Andressa P. Stolz, Macarthur Vilanova, Guilherme Watte, Antonio N. Kalil

**Affiliations:** 1 Santa Casa de Misericordia de Porto Alegre, Porto Alegre, Brazil; 2 Universidade Federal de Ciencias da Saude de Porto Alegre, UFCSPA, Porto Alegre, Brazil; 3 Universidade Federal de Santa Maria, UFSM, Santa Maria, Brazil; 4 Universidade Católica de Pelotas, UCPEL, Pelotas, Brazil; 5 Hospital Universitário de Canoas, Canoas, Brazil; 6 Hospital Sao Vicente de Paulo, Passo Fundo, Brazil; 7 Universidade Regional Unijui, Ijui, Brazil; 8 Universidade Federal do Pampa, Unipampa, Uruguaiana, Brazil; 9 Universidade do Vale do Taquari, Univates, Lajeado, Brazil; 10 Círculo Operadora Integrada de Saúde, Caxias do Sul, Brazil; 11 Universidade de Santa Cruz do Sul, UNISC, Santa Cruz do Sul, Brazil; 12 Brigada Militar do Rio Grande do Sul, Porto Alegre, Brazil; Universidade Federal da Bahia, BRAZIL

## Abstract

**Background:**

Limited data is available regarding the frequency of COVID-19 in populations that are highly exposed to SARS-CoV-2. In this cross-section study we evaluated COVID-19 seroprevalence in military police forces of 10 major cities in Rio Grande do Sul, South of Brazil.

**Methods:**

Sampling was randomly performed in clusters, in respect to the number of professionals at service per city and military unit. Research subjects were evaluated on July 23, 2020 (first wave peak in Brazil). Clinical information was obtained, and venous blood was taken for ELISA testing (IgA, and IgG antibodies). Sample size consisted of 1,592 military workers (33.6% of study population). They were mostly man (81.2%) and young (median 34 years-old). Most had been asymptomatic (75.3%) during pandemic, and 27.5% reported close contact with COVID-19 cases (after a median time of 21 days). Antibodies were detected in 3.3% of the participants, mostly IgA (2.7%), and IgG (1.7%). After 3 weeks, 66.7% of IgA and IgG results turned negative, in addition to 78.3% and 100% of borderline IgA and IgG results, respectively.

**Conclusion:**

The seroprevalence of COVID-19 amongst military police was at least 3.4 higher than the findings of other studies performed in the general population, in the same cities and dates. Most detectable antibodies were of IgA class, which implies recent exposure. Asymptomatic people were more prone to have negative antibody titters in the second run.

## Introduction

Brazil has been hardly hit by the COVID-19 outbreak, the first case of COVID-19 was confirmed by the Minister of Health, on February 25, 2020, in a Brazilian traveler returning to São Paulo from a trip through northern Italy. At the end of July, Brazil was facing first wave peak with 2,662,485 confirmed cases and 92,475 deaths due to the new coronavirus. Rio Grande do Sul, the southernmost state in Brazil where our study was conducted, reported the first case of COVID-19 on February 29, 2020. On July 31, 66,692 cases and 1,876 reported deaths had been reported [1–3]. Despite that, the epidemiology of COVID-19 has been poorly described in the country, and most investigations have tested the general population with immunocromatographic tests [2, 4].

Here we performed a seroprevalence study in the military police force of ten cities in Rio Grande do Sul, South of Brazil. Participants were all evaluated using ELISA testing, and serology was repeated for positive cases three weeks after initial evaluation. Both IgG and IgA antibodies were detected, since these are one of the main effector molecules against viruses. The choice for IgA in favor of IgM was based on previous data showing IgA detection in 75% of COVID-19 patients within the first week, in addition to more persistent response in comparison to IgM [5].

The COVID-19 pandemic affects different occupational activities in several countries, including Brazil, but the available data and research of such impact in relation to the frequency of COVID-19 in populations highly exposed to SARS-CoV-2, such as the military police are still limited. For this reason, it is essential to have data on these populations, especially in Latin America, where there is a major focus of the disease [6].

## Methods

This was a cross-sectional study involving military police in ten cities of Rio Grande do Sul: Porto Alegre, Caxias do Sul, Canoas, Pelotas, Santa Maria, Passo Fundo, Uruguaiana, Santa Cruz do Sul, Ijui, and Lajeado. A total of 4,777 military policeman were at service in these places. Forty-five individuals were excluded from the seroprevalence study because of previously confirmed COVID-19 (0.9%), resulting in 4,732 professionals for analysis. A sample of 1,592 subjects was calculated (33.6% of study population) considering estimated prevalence levels of 3.0% and 8.0%. Sampling respected the proportionality of each 28 military regiments belonging to the 10 municipalities. This sample was not intended to be representative of the State’s population of military police force. In case there was a refusal at a regiment, research participant was replaced by the next person drawn from the list of the respective regiment.

Research subjects were evaluated over a period of 3 days, starting on July 23, 2020. Clinical and demographic data were obtained from the each of the participants, using digital platform REDCap. Venous blood was taken in 5 mL EDTA tubes with separator gels from all participants, for COVID-19 IgA and IgG antibody detection, performed with Enzyme-Linked Immunosorbent Assay (ELISA) testing, following manufacturer’s instructions (EUROIMMUN^®^). The test is considered to be of high sensitivity and specificity to detect immunoglobulins against SARS-CoV-2.

Continuous variables were expressed as the mean, standard deviation or median (interquartile range) and were compared with the Mann–Whitney *U*-test. Categorical variables are expressed as numbers (%) and were compared by Chi-square test or Fisher’s exact test, as appropriate. A P value of less than .05 was considered statistically significant. Statistical analyses were performed using SPSS software, version 22.0. Participation in the study was voluntary and required signed consent of the research subject, whose identity was preserved at all times. The ethical approval was obtained from The Brazilian National Ethics Committee (process number CAAE: 31689120.2.0000.5335).

## Results

A total of 1,592 military police individuals were evaluated. Subjects were mostly man (81.2%) and median age was 34 years-old (± standard deviation, 8 years-old). According to military ranks, these were 1,233 soldiers (82.1%), 162 sergeants (10.8%), 48 lieutenants (2.9%), 32 captains (2.1%), and 117 other commissioned officers (2.1%). Most reported no chronic medical conditions, except for high blood pressure (3.1%) and asthma (2.6%). The vast majority reported use of personal protective equipment at work, such as masks (99.2%), gloves (23.2%), and face shields (9.6%). Most had been asymptomatic (75.3%) during pandemic, while 13.7% had cough, 8.2% fever, and 2.6% dyspnea. A total of 438 individuals (27.5%) informed close contact with COVID-19 cases (median time for exposure was 21 days before study participation). Up to the date of randomization (June 30^th^, 2020), 45 participants (0.94% of total) were diagnosed with COVID-19 and as per protocol these were excluded from the seroprevalence analyses. Three of these policeman (6.7%) required hospitalization and no death was reported.

**Fig 1:**
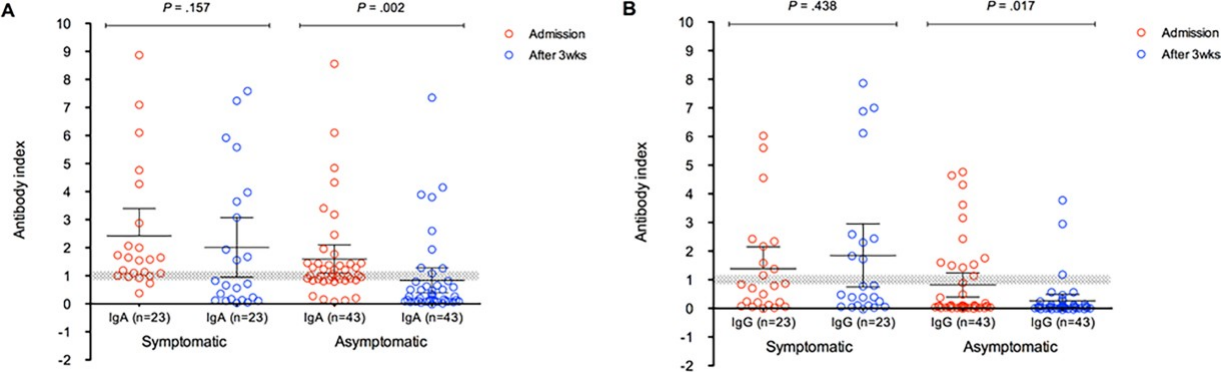
Seroprevalence of COVID-19 in the military police of Rio Grande do Sul.

Overall, antibodies were detected in 3.3% of participants, some had both antibodies positive, these were mostly of IgA class (2.7%), and 1.7% of participants had IgG. In addition, some individuals had antibody indexes in the borderline zone: IgA 1.7%, IgG 0.3% (Table 1). Presence of symptoms during pandemic was more frequent in individuals demonstrating IgA antibodies (n = 17; 4.4%), in comparison to asymptomatic workers (n = 26; 2.2%) (p = 0.023). Similar results were observed for IgG, regarding presence of symptoms (n = 12; 3.1%), in comparison to those who did not have symptoms (n = 16; 1.3%) (p = 0.023). Report of close contact with COVID-19 cases was also associated with the presence of IgA antibodies: 18 (4.2%) vs. 25 (2.2%), respectively (p = 0.028); IgG detection was also associated with exposure to a confirmed COVID-19 case: 15 (3.4%) vs. 13 (1.1%), respectively (p = 0.002). In the second phase of study (3 weeks after initial evaluation), 66.7% of IgA and IgG results turned negative, in addition to 78.3% and 100% of borderline IgA and IgG results, respectively. Of the 36 participants who tested positive for IgA in the first analysis, 24 became negative in the second analysis (66.67%). Of the total of 21 study participants who tested positive for IgG in the first analysis, 14 became negative in the second analysis (66.67%). Of the 36 participants who initially tested positive for IgA in the first analysis, it was observed that 7 of these became IgG positive (19.44%) (Table 1). Conversely, considering the 21 participants who tested positive for IgG in the first analysis, 8 of these (38.10%) tested positive for IgA in the second test. In Fig 1 it is possible to observe the variation of the IgA and IgG antibody in the first day of the collection and 3 weeks later, showing that asymptomatic people were more prone to have negative antibody titters in the second run (Fig 1). This shows that our results are important as they help to better understand the epidemiology and impact of COVID-19 in a population exposed daily to the virus.

**Table 1:** Marked variability in COVID-19 seroprevalence according to the city of origin in a survey of military police workers in the South of Brazil.

City	IgA positivity	IgG positivity
Passo Fundo (n = 101)	7 (6.9%)	5 (5.0%)
Canoas (n = 129)	6 (4.7%)	3 (2.3%)
Caxias do Sul (n = 116)	6 (5.2%)	0 (0.0%)
Uruguaiana (n = 41)	2 (4.9%)	1 (2.4%)
Ijuí (n = 25)	1 (4.0%)	1 (4.0%)
Porto Alegre (n = 695)	18 (2.6%)	15 (2.2%)
Lajeado (n = 123)	2 (1.6%)	2 (1.6%)
Santa Maria (n = 177)	1 (0.6%)	1 (0.6%)
Santa Cruz do Sul (n = 59)	0 (0.0%)	0 (0.0%)
Pelotas (n = 126)	0 (0.0%)	0 (0.0%)
**Total:** 1,592 participants	43 (30.5%)	28 (18.1%)

Note: Borderline results were not considered for the purpose of these analyses.

All military workers presenting with IgA antibodies (either positive or borderline) were referred to a medical doctor and preventively isolated from the troop. PCR was requested for 71.4% of these individuals (n = 50), and four were found to be PCR-positive (8.0%).

## Discussion

Despite the urgent need for population-based data on COVID-19, few epidemiological surveys had been conducted in Brazil, by the time of writing of this manuscript [2, 4, 5, 7, 8]. COVID-19 affects different occupational activities, and with a fragile healthcare system, social and economic issues in Latin America affect the entire situation. In Brazil, COVID-19 is infecting more and more people, with 2.5% of lethality [1, 3]. The Military Police has an important role, with the purpose of protecting the citizen, society and public and private property, preventing criminal offenses and administrative infractions. In addition to the constitutional duties, in Brazil, military police perform several other tasks that influence people’s daily lives, collaborating with all segments of the community, reducing conflicts and generating a sense of security [9]. In this study, we demonstrated that 27.5% of military policemen had close contact with confirmed COVID-19 cases in ten major cities in Southern Brazil, at the first wave peak in 2020. Moreover, 3.3% had detectable antibodies against SARS-CoV-2, which is at least 3.4 higher than the findings of other studies performed in the general population, in the same Brazilian cities and dates [2, 7]. Considering this, and the fact that borderline results were not considered in the definition of ELISA positivity, the 3.3% number is possibly underestimated, and the real frequency of COVID-19 in this highly exposed population might be actually higher. In a study performed in densely infected areas of São Paulo (Southeast Brazil) using chemiluminescence immunoassay, seroprevalence of COVID-19 was 4.7% on May 4 and May 12, 2020 [8].

It is interesting to note that the first COVID-19 case was reported in Brazil on February 26, 2020. In Rio Grande do Sul state, the first confirmed COVID-19 occurred in March 10, in a traveler returning from Europe. Despite the occurrence of limited number of cases, on April 1^st^ the governor of Rio Grande do Sul applied strict restrictions to local businesses. A large proportion of people remained at home, except for critical jobs such as health care and police forces. Therefore, this study evaluated a population that remained at work at all times during COVID-19 pandemic, mostly using personal protective equipment at work. The finding that most antibodies detected in the study (on July 23) were of IgA subclass suggest that infection was acquired in the couple of weeks before study initiation, at the first epidemic COVID-19 wave peak in Rio Grande do Sul. COVID-19 was not severe in our population, composed mostly of healthy young man with no underlying medical conditions. No death was reported in the study. There is a study in the literature that also investigated the seroprevalence of COVID-19 in the state of Rio Grande do Sul, including the cities evaluated also in our study. An infection rate of up to 20% was found, considering the total of 4,500 people (total population and not just military police). These data corroborate the findings of our study as they alert the high transmission rates of SARS-COV-2 [9].

It is also worth noting that about two thirds of IgA and IgG antibodies turned negative after three weeks of follow up, particularly in patients who have been asymptomatic during pandemic. Similar findings have been reported by others [10–12]. In one investigation, 40.0% of the asymptomatic seropositive individuals turned negative after a short period of time (8 weeks after hospital discharge), while 12.9% of people in the symptomatic group became negative for IgG at the beginning of the convalescence phase. In addition, asymptomatic people exhibited more discrete levels of pro and anti-inflammatory cytokines, in comparison to symptomatic patients. Together, these findings suggest that asymptomatic individuals have a lower viral load, and a milder immune response to SARS-CoV-2 infections [13, 14]. The impact of antibody disappearance in host protection against COVID-19 remains to be better elucidated, since additional immune mechanisms may probably play a role. This findings may be of great relevance for considerations regarding COVID-19 vaccine use, as well as for the conduction of epidemiological studies.

There are some limiting points for the study. As this was a cross-sectional study, it was not always possible to establish temporal associations and data on variables related to the epidemiology of SARS-CoV-2 are lacking. Despite that, our findings show important results, including the documentation of high levels of contamination in Southern Brazil during first wave peak in 2020.

In conclusion, this study presents the most solid and updated seroprevalence data for a population of highly exposed individuals to COVID-19 in Brazil, regarding first wave peak in 2020. Predominance of IgA antibodies in the study suggested recent exposure to SARS-CoV-2. Most IgA and IgG antibodies disappeared after three weeks in the study, particularly in asymptomatic individuals.
